# Special Issue: “Digestive Inflammation and New Therapeutical Targets”

**DOI:** 10.3390/ijms25084361

**Published:** 2024-04-15

**Authors:** Alain Couvineau, Cécile Haumaitre

**Affiliations:** INSERM UMR1149/Inflammation Research Center (CRI), Faculty of Medicine X. Bichat, Université Paris Cité, 75018 Paris, France

Inflammatory diseases commonly associated with humans are chronic inflammatory gastrointestinal diseases (CIGDs). CIGDs may affect any part of the gastrointestinal tract, but they are most commonly associated with the liver, pancreas, bile duct, intestines, and colon, as well as the stomach and esophagus. A number of diseases are associated with CIGDs, including hepatitis, pancreatitis, cholangitis, intestinal bowel diseases (IBDs), gastritis, and esophagitis. The etiology, mechanism, and symptomatology of CIGDs differ based on the part of the organ affected. Many of these conditions share similar characteristics, including a dysregulated immune system, imbalances between pro- and anti-inflammatory processes, disruptions of epithelial barrier function, and the involvement of microbiota or viruses. Aside from the chronic nature of these inflammations, CIGDs are also important risk factors for developing carcinogenesis in numerous organs, including the stomach, liver, esophagus, bile ducts, pancreas, and colon, in which these cancers are generally prevalent. Although the mechanisms underlying this relationship have only partially been explored, several possible mechanisms have been identified that may contribute to the association between inflammation and cancer. The chronic inflammatory environment has several effects on genotoxicity, in which the presence of inflammatory cell products may lead to genetic and/or epigenetic alterations [1,2,3,4,5]. The cell death and proliferation induced by CIGDs may increase the risk of mutations, while inflammation-induced cell death leads to compensatory proliferation [1,2,3,4]. Moreover, it was observed that a deregulated digestive microbiota occurs in chronic inflammation and increases cancer risk, particularly in colorectal and liver cancers [5,6,7,8,9,10,11]. It is also known that chronic inflammation is associated with the development of gastric and liver cancers in the presence of infectious agents such as Helicobacter pylori or the hepatitis C virus [3,5,6,12]. In this context, the development of a new and innovative therapeutic arsenal for the treatment of CIGDs remains a key challenge. This Special Issue, dedicated to “Digestive Inflammation and Emerging Therapeutic Targets”, features several articles addressing this evolving challenge, each briefly summarized below.

Sexual dimorphism is a prevalent feature observed in numerous common disorders. However, the impact of gender on inflammatory susceptibility remains ambiguous. In an attempt to better understand the role of sex in inflammatory bowel disease (IBD), Cassado-Bedmar et al. explored the sex differences in the IL-10 anti-inflammatory pathway response and their impact on the microbiota. The authors demonstrated sex differences and sex-associated inflammatory susceptibility in colitis using an animal model invalidated for the IL-10 cytokine (IL-10^–/–^). The characterization of the colonic and fecal inflammatory phenotypes showed that the female mice had a higher susceptibility to developing colitis compared to the male mice. Specifically, the authors showed that female mice had differences in their microbiota, such as a higher abundance of the Bacteroidetes phylum and a lower presence of the Firmicutes phylum. This highlights the importance of carefully selecting the sex of animals in experimentation, considering the unique characteristics associated with each gender. This study significantly contributes to the understanding of sex differences in colitis development in mice and IBD patients.

The gut microbiota (GM) and its dysregulation play a crucial role in the pathogenesis of various gastrointestinal diseases. Colon diverticulosis and its clinical manifestations (diverticular disease (DD)) are one of the most important digestive disorders among intestinal diseases. Tursi et al. outlined in their review article the significant role of GM dysbiosis in DD and compared it with the available data on dysbiosis observed in IBD. The authors also discussed the potential of probiotics in restoring dysbiosis in DD.

Among treatments, Infliximab (IFX) has tremendously enriched the therapy of inflammatory bowel diseases (IBDs) and other immune-mediated diseases. IFX is a monoclonal antibody directed against TNFα, a major inflammatory cytokine in IBD, and is widely used in the treatment of Crohn’s disease and ulcerative colitis (UC). However, the IFX treatment displayed a high degree of variability in efficacy between patients and a loss of efficacy over time. In this issue, Blais et al. used the DSS-treated mouse model known for developing UC and demonstrated that OxA treatment enhanced mucosal healing. It also reduced colonic myeloperoxidase activity and circulating levels of lipopolysaccharide-binding protein, IL-6, and TNFα, while decreasing the expression of cytokine-encoding genes in colonic tissues. Notably, OxA exhibited superior efficacy compared to IFX. This study provides evidence that orexins may be a promising alternative therapy for UC.

One of the most common complications of IBD is intestinal fibrosis, characterized by uncontrolled extracellular matrix deposition, leading to complications that require surgical intervention. The key player in the fibrogenesis process was identified as transforming growth factor (TGF), which could be modulated by various molecules. Among these, peroxisome proliferator-activated receptor (PPAR-γ) and its agonists showed notable antifibrotic effects. In this issue, Pompili et al. demonstrated an increase in epithelial-mesenchymal transition (EMT) and senescence signal activation in IBD patients compared to control subjects. The same observations were shown in the mouse model of DSS-induced colitis. In addition, GED, a PPAR-γ agonist, reduced all pro-fibrotic pathways, to some extent, more efficiently than the gold standard 5-ASA used in IBD treatment. These findings suggest that PPAR-γ activation may be beneficial for IBD patients. 

The investigation of non-coding RNAs (ncRNA) has unveiled new insights into gene expression regulation in the last few years. Among ncRNA, the long non-coding RNA (lncRNA) has been implicated in various diseases. The present review presented by Triantaphyllopoulos K. summarizes the current knowledge of lncRNAs in cellular function, with a focus on their role in the pathogenesis of IBD in humans (ulcerative colitis (UC) and Crohn’s disease (CD)), as well as Johne’s disease (JD) in cattle, caused by Mycobacterium avium subspecies partuberculosis (MAP). The focus is on the role of recently characterized and novel lncRNAs in IBD and JD, with the aim of elucidating the associated genes and pathways and identifying relevant potential biomarkers of these diseases in cattle and humans.

In the context of inflammatory pancreatic diseases such as pancreatitis, the high plasticity of adult pancreatic acinar cells is needed for pancreatic regeneration. Pancreatic acinar cell reprogramming leads to the transformation of acinar cells into duct-like cells by a pancreatic acinar-to-ductal metaplasia (ADM) process. However, if the stimuli causing ADM persist, as in chronic pancreatitis or in the presence of oncogenic signaling, ADM is irreversible and can lead to the development of a precancerous lesion called pancreatic intraepithelial neoplasia (PanIN), a precursor to pancreatic ductal adenocarcinoma (PDAC). The present review by Mastrand-Daucé et al. was devoted to the state of knowledge on the cellular and molecular aspects of ADM. Clearly, the ultimate understanding of the cellular and molecular mechanisms involved in ADM is essential not only for the development of novel therapies targeting pancreatitis but also for early detection and the development of innovative treatments for pancreatic cancer.

In conclusion, CIGDs present a range of gastrointestinal disorders, posing challenges in understanding their causes and treatment. These diseases share common features such as dysregulation of the immune system, disruption of epithelial barriers, cellular reprogramming, chronic inflammation, and higher cancer risks. Studying factors influencing susceptibility and investigating cellular and molecular mechanisms, as demonstrated by the studies highlighted, offer promising avenues for improving our understanding and developing more effective therapeutic approaches to CIGDs.
